# Determinants of health volunteer training in natural hazard management in Iran

**DOI:** 10.4102/jamba.v15i1.1384

**Published:** 2023-06-23

**Authors:** Fereshteh F. Amini, Alireza A. Hidarnia, Fazlollah F. Ghofranipour, Mohammad E. Motlagh, Abdul Majid RahPima, Navvab Shamspour

**Affiliations:** 1 Department of Medical Surgical, School of Nursing and Midwifery, Tehran University of Medical Sciences, Tehran, Islamic Republic of Iran; 2 Health Education and Health Promotion, Faculty of Medical Sciences, Tarbiat Modares University, Tehran, Islamic Republic of Iran; 3 Department of Health Education and Health Promotion, Faculty of Medical Sciences, Tarbiat Modares University, Tehran, Islamic Republic of Iran; 4 Department of Community Medicine, School of Medicine, Ahvaz Jundishapur University of Medical Sciences, Ahvaz, Islamic Republic of Iran; 5 Department of Public Policy, Islamic Azad University, Tehran Branch, Iran; 6 Research Center for Emergency and Disaster Resilience, Red Crescent Society of Islamic Republic of Iran, Tehran, Islamic Republic of Iran

**Keywords:** education, healthcare volunteer, preparation, natural education, natural hazard, hazard management

## Abstract

Both natural and man-made dangers cause bodily harm, as well as social, economic and environmental harm. In order to minimise the complications of these threats, proper training and preparedness are crucial. The purpose of this study was to look at the factors that affect how well-trained healthcare volunteers are for natural hazards in Iran. Using the Preferred Reporting Items for Systematic Reviews and Meta-Analyses approach, a systematic review of literature on the factors influencing the training of healthcare volunteers in natural hazard published between 2010 and 2020 was conducted. The Google Scholar search engine, PubMed (Medline and Central), Science Direct and Web of Science databases were searched using both individual and combined key phrases. The Strengthening the Reporting of Observational studies in Epidemiology checklist was used to select and evaluate 592 observational and quasi-experimental articles. Finally, the study comprised 24 papers that satisfied the research criteria and made good use of good technique, sample size and adequate tools for validity and reliability. The most useful variables for disaster preparedness were job self-efficacy, strategic decision-making and quality of work-life, job performance, job motivation, knowledge, awareness and health literacy.

**Contribution:**

To avoid calamity, a thorough training program is required. Therefore, the most crucial objectives for health education specialists are to identify the factors that determine disaster preparedness, train volunteers and provide fundamental techniques to reduce natural dangers.

## Introduction

Both naturally occurring and man-made physical disasters can cause human casualties, societal and economic harm and environmental destruction. Losses and injuries to people, places and the environment result from being vulnerable to these accidents (Bizid et al. 2015). Natural disasters have expanded awareness of disasters and what can be done to prevent them on a national and worldwide scale, as have the concerns posed by climate change (such as high summer heat, winters without snow, floods and droughts) (Kipp 2003). Natural disasters were the primary cause of fatalities from 1990 to 2015, and 2008 was the most disastrous year with more than 500 incidents, according to the World Health Organization (WHO) (Nahayo et al. 2018). More than a million people per year died from natural disasters throughout the first half of the 20th century, with earthquakes, floods and droughts being the main causes of death.

Asia suffered the most from the 396 natural disasters that were reported in crisis management statistics in 2019, which resulted in 11755 fatalities, 95 million injuries and 103 billion dollars in economic losses (2019 Global Natural Disaster Assessment Report 2020).

The WHO’s catchphrase for 2009 was ‘Disaster Health’, and the group has vowed to teach its members in disaster management planning, management and coordination (Farajzadeh et al. 2017). About 70.2 million (53.3%) of the 131.7 million individuals who needed health care globally in 2018 lived in the Eastern Mediterranean region, and roughly 80 million of them were impacted by natural catastrophes and armed conflict (Asghari, Banaei & Miralanogh 2016). Iran ranks sixth internationally and fourth in Asia in terms of the frequency of natural disasters, necessitating public education on disaster management (Farajzadeh et al. 2017). Historical devastating earthquakes in Iran with magnitudes more than 7 (Shahr-e Rey, Buin-e Zahra, Bam, etc.) show that earthquakes are still among the most deadly natural phenomena in Iran.

The number of catastrophic disasters has significantly increased in recent decades, primarily because of social and economic factors like population growth and migration, industrial development and human intervention in natural systems. This is especially true because communities are becoming more vulnerable (UNISDR Strategic Framework 2016–2021). According to a 2018 study, Tehran, the most populous city in Iran with a population of around 8.9 million and an area of roughly 615 square kilometers, is particularly vulnerable to natural disasters like earthquakes, with regions 5, 4, 15 and 20 being the most at risk (Najafi Ghezeljeh et al. 2019).

Potential damages from disasters are frequently difficult to estimate. However, these harms can usually be quantified because of knowledge of frequent risks, demographic trends and socioeconomic progress (Ahmadi & Manoochehri 2020). According to estimates, developing nations account for more than 90% of the victims of natural disasters worldwide. These nations experience more than 20 times more earthquake deaths than developed nations. Although crises cannot be avoided, they can be mitigated, and communication networks can play a crucial role in delivering relief and support to the injured (Tint, McWaters & Driel 2015). The predisaster activities that can lessen the effects of disasters, such as casualties, injuries, illness and other negative effects on the body, mind and community, damage to property, loss of services and all social, economic and environmental degradation, include emergency preparedness, training programs, maneuvers and appropriate public response. Innovations are needed to minimise catastrophe risk by 2030, shift the paradigm of public education and knowledge of disaster response and assist decision-makers in improving disaster planning and response while restoring normalcy (Parajuli 2020). One of the most effective strategies for motivating an audience to help manage the crisis process in any situation is the voluntary participation of individuals in disaster planning and response, relief efforts and restoration (Yu, Yang & Li 2018). The general population will learn more readily if the instruction is made simpler. People desire to participate actively in their education and be educated using images. Listening does not result in actual learning; it merely broadens one’s knowledge. Training often involves a number of main techniques, including educational boards, billboards and posters, roadmaps, hands-on activities and questions and answers (Pollack 2020). Manoeuvres should be carried out and reviewed to make sure that yearly manoeuvres are necessary to prepare for natural catastrophes in order to make sure that families have gotten all the training. With today’s modern computer systems, wide media networks and early warning systems, it is feasible to become aware of the threat of natural disasters sooner. However, in order for the disaster alert system to be effective, people must be educated so that they have the necessary information beforehand and can react effectively in an emergency (Pollack 2020).

The Iranian healthcare system is looking for ways to raise people’s health literacy and awareness so they can respond to disasters in the best and safest way possible by leveraging their expertise.

In Iran, volunteers play a critical part in the Red Crescent Society’s first aid services. It is critical to understand and take into account the educational demands of these volunteers (Kebriyaii, Hamzehei & Khalilzadeh 2021; Khatami et al. 2010; Ramezani Nejad & Alaedin 2009).

One of the top goals of the Iranian Ministry of Health is disaster risk management, which was also stressed in the second priority of the Sendai document (disaster risk management in countries’ plans and strategies) in 2015.

Therefore, it is essential to establish a fresh, comprehensive training program that can be applied on a big scale in a country like Iran, which is vulnerable to calamities and unforeseen events.

This necessity and the reality that even in Iran’s most disaster-prone regions, disaster preparedness levels are still only moderately high and pose the fundamental dilemma of inspiring people to take preventive action and to continue receiving health education.

The researcher’s main focus is on providing the best incentives and environment for preventative activities, health education and its continuation in society.

As a result, the current systematic review was undertaken to learn more about the variables influencing the education of healthcare volunteers in Iran during natural disasters.

## Information and methods

Using the Preferred Reporting Items for Systematic Reviews and Meta-Analyses (PRISMA) approach, a systematic review of descriptive analytical, cross-sectional and semi-experimental English and Persian literature published between 2010 and 2020 on the topics of the determinants affecting healthcare volunteers’ training in natural disasters was conducted (Moher et al. 2009).

The Google Scholar search engine, PubMed (Medline and Central), Science Direct and Web of Science databases were searched individually and in combination for the terms ‘Disaster Education’, ‘Disaster Preparedness’, ‘Crisis Preparedness’ and ‘Disaster Risk Reduction’.

The references of the discovered articles were also assessed for potential future research, and relevant research was incorporated into the study.

The full texts of 592 observational and quasi-experimental publications published between 2010 and 2020 that used the terms ‘Disaster Education’, ‘Disaster Preparedness’, ‘Crisis Preparedness’ and ‘Disaster Risk Reduction’ as their primary variables were chosen.

The same articles (112 titles) were deleted after analysing their titles and abstracts. The remaining 480 titles underwent a thorough evaluation, and 320 titles were eliminated for lack of topical relevance. Inclusion criteria were the quantitative studies in which ‘disasters’ and ‘preparation of healthcare volunteers’ were one of their main variables, studies with the available full text, and studies conducted between 2010 and 2020. Exclusion criteria were low score of the studies based on the Strengthening the Reporting of Observational studies in Epidemiology (STROBE) checklist, studies with unsuitable quality in terms of methodology, sample size, appropriate tools and necessary data and studies that were conducted before or after the determined time period of this study and systematic reviews.

One hundred and sixty other articles complied with the STROBE criterion.

Cohort, case control and cross-sectional study information is reported using this checklist, which comprises 22 items (Vandenbroucke et al. 2007).

Eighty two papers were eliminated because of poor study quality, and 54 articles were eliminated because of a lack of material required by the research standards, per the checklist’s findings.

**FIGURE 1:**
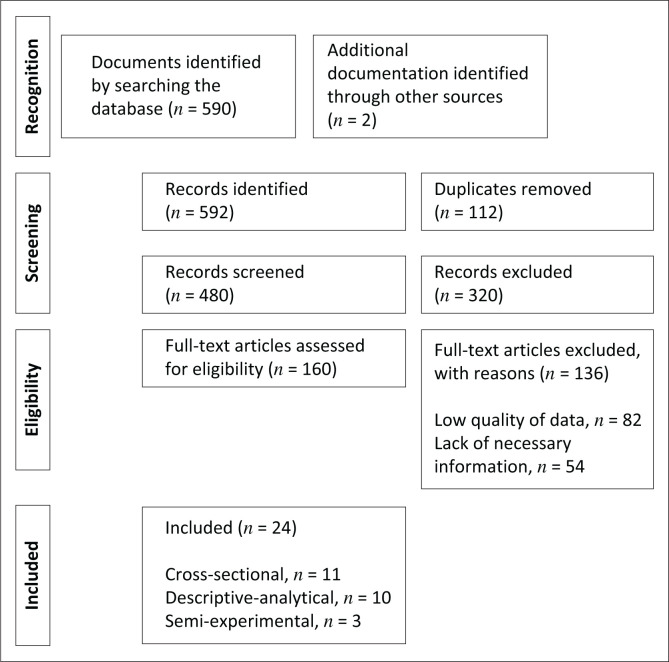
Flowchart of steps for entering a systematic review of preferred reporting items for systematic reviews and meta-analyses.

Finally, the study contained 24 papers that satisfied the research criteria – used good methodology, an appropriate sample size and reliable and valid methods (Figure 1).

## Findings

Public participation and preparedness have been found to be crucial in lowering the risks of disaster in almost all studies.

Promoting awareness and expanding knowledge can help to lessen the damage that disaster risk causes. Table 1 lists the features of the 24 included articles under the headings of disaster education, disaster preparedness and disaster information.

**TABLE 1:** Details of studies related to the effect of education on disaster preparedness.

Row	Authors	Subject	Year and country	Study type and sample size	Result
1	Ahadpourmarin and Shahrakipour (2013)	Investigating the effect of training Red Crescent workers using new technology against natural disasters in Tehran from the perspective of workers of the Relief and Rescue Organization	2013 (Iran)	Cross-sectional study (*n* = 110)	Proper training of human resources, especially rescuers, and equipping them with new technology have positive effects on reducing disaster casualties.
2	Farajzadeh et al. (2017)	Preparedness of nurses for crises and disasters in Imam Khomeini and social security hospitals of Saqqez	2014 (Iran)	Cross-sectional study (*n* = 257)	There is a significant relationship between knowledge with age and function with gender and management experience. The nurses’ preparedness score was average and unfavourable. So training program should be implemented for them by creating the appropriate motivation and conditions for disaster preparedness.
3	Jahangiri et al. (2014)	Selected people’s strategies in information and public education for earthquake preparedness: a survey of the perspectives and expectations of the people of Tehran	2014 (Iran)	Cross-sectional study (*n* = 211)	The information does not change people’s attitudes and behaviors; however, appropriate measures should be taken to encourage people to adopt preventive behaviors while increasing their understanding of the dangers of earthquakes. The choice of information methods and educational tools should be in accordance with the selected methods of the community and the indigenous culture of each region.
4	Asghari Zamani and Babaie Aqdam (2016)	Evaluating the level of efficiency of the road network during unexpected events in Tabriz city	2017 (Iran)	Descriptive analytical study (16 networks in Tabriz)	If the city’s communication network is not damaged after the disasters, the casualties will be greatly reduced. The condition of most road networks in Tabriz city is unfavourable and lacks the necessary efficiency.
5	Najafi Ghezeljeh et al. (2019)	Effect of education using the virtual social network on knowledge and attitude	2019 (Iran)	Semi-experimental study (*n* = 60)	Providing disaster preparedness training sessions through virtual sessions has increased the effectiveness of learning and promoted a positive attitude of emergency nurses towards disaster preparedness.
6	Kolivand et al. (2021)	Benchmarking rate of members of the crisis and disaster management committee in Iranian public hospitals	2020 (Iran)	Cross-sectional study (*n* = 410)	Improving organisational performance is possible with optimisation tools. The findings showed that training courses should promote optimisation to improve disaster management performance.
7	Nakhaei et al. (2019)	The effect of educational intervention on nurses’ preparedness in emergencies and disasters	2019 (Iran)	Semi-experimental study (*n* = 75)	The average scores of the ability and attitude of the leading forces in the face of disasters increased through the training intervention.
8	Ostadtaghizadeh et al. (2016)	Health Consequences and Management of Explosive Events	2017 (Iran)	Descriptive analytical study	Whether natural or artificial, explosive events have the highest casualties, and the knowledge of its prevention, protection and management principles and its simulated training program is necessary.
9	Hosseini, Safarnia and Poursaeed (2016)	The relationship between knowledge management related to earthquake and social factors of resiliency (case study: Trained Volunteers in Neighborhood of Sarasiab in Kerman)	2017 (Iran)	Descriptive analytical study (*n* = 140)	Resilience is an approach to reduce casualties, and knowledge and awareness effectively promote resilience. The average knowledge management is not different from gender groups, but knowledge management is different from age and educational groups.
10	Heidarnia et al. (2016)	Effect of a web-based educational program on prevention of tobacco smoking among male adolescents: an application of prototype willingness model	2017 (Iran)	Descriptive analytical study (*n* = 114)	Training strategy is especially important for risk prevention.
11	Liou et al. (2020)	Relationships between disaster nursing competence, anticipatory disaster stress and motivation for disaster engagement	2020 (Taiwan)	Cross-sectional study (*n* = 90)	Anticipatory disaster stress was positively related to nurses’ competence and motivation.
12	Lee and Lee (2019)	Disaster awareness and coping: Impact on stress, anxiety and depression	2018 (Korea)	Cross-sectional study (*n* = 291)	This study showed that disaster awareness, understanding of disaster response strategy and disaster information level directly reduce anxiety and depression.
13	Patel et al. (2020)	A socioeconomic-based analysis of disaster preparedness, awareness and education	2020 (USA)	Cross-sectional study (*n* = 111)	University students are significantly different from other students in disaster risk reduction (DRR) education. Education and preparation were related to life and their ethnic background.
14	Berhanu et al. (2016)	Experiences and training needs of health professionals about disaster preparedness and response in southwest Ethiopia	2016 (Ethiopia)	Cross-sectional study (*n* = 377)	The majority of people have poor knowledge of disaster alert indicators and cited the need for disaster preparedness and management training.
15	Tint et al. (2015)	Games for learning and dialogue on humanitarian logistics: Applied improvisation training for disaster readiness and response; preparing humanitarian workers and communities for the unexpected	2015 (Philippines)	Cross-sectional study	Artificial intelligence has many benefits, including creating satisfaction among rescuers, making timely strategic decisions, removing cultural and linguistic barriers and creating disaster preparedness.
16	Khalili, Rashidy and Pirdashti (2018)	Considering the role of public participation to improve urban crisis management and its effective elements are based on Ragers’ public participation theory (case study: Behshar city)	2018 (Iran)	Descriptive analytical study (*n* = 264)	The role of public participation is effective in improving disaster management performance. The success of a program depends on the role of the people, and the identification of needs and risk reduction measures is possible only with the participation of the people.
17	Azmi et al. (2017)	Investigating the role of indigenous people in understanding natural hazards and preparedness against them at Zalu Ab Rural District, central district of Rawansar township, Kermanshah province	2017 (Iran)	Descriptive analytical study (*n* = 150)	The greater the power of predicting disasters, the greater their self-confidence and the urgent need for knowledge and awareness for personal preparation.
18	Farouji and Bahramian (2021)	Vulnerability zoning of Tehran in natural disasters concerning population groups	2021 (Iran)	Cross-sectional study (population groups in the 22 districts of Tehran)	The 4, 5, 15 and 20 areas of Tehran are priority areas that need to be capacity building to empower vulnerable groups.
19	Motieei Langroudi et al. (2015)	Investigating the role of participatory management in reducing flood effects (case study: Villages of Zangmar Mako river basin)	2015 (Iran)	Descriptive analytical study (*n* = 292)	Participatory management effectively reduces the effects of floods. In all aspects, the participation of villagers and officials is important, which shows the importance of prevention and risk management in coping with floods.
20	Kaveh-Firouz et al. (2009)	Attitudes of the people of Tehran towards the earthquake	2011 (Iran)	Semi-experimental research (*n* = 1600)	Even though people have considered the earthquake somewhat serious, their awareness about its coping and prevention is low.
21	Seyedin et al. (2019)	Disaster coping planning: an effective approach to disaster risk reduction in Iranian health organisations	2011 (Iran)	Cross-sectional study (*n* = 102)	Measures should be taken to improve the disaster management system, such as re-engineering to design the disaster management system, such as preparation activities, standards and protocols, staff training and regular manoeuvers.
22	Porfande and Shoqi (2016)	Importance and effect of disaster management programs on the level of preparedness of nursing staff of the Imam Reza Hospital in Bojnourd	2016 (Iran)	Descriptive analytical study (*n* = 41)	The results reflected the positive effects of disaster management training courses on nursing staff.
23	Hayavi Haghighi et al. (2019)	National Information Systems of natural crises in some countries	2019 (National Information Systems of Natural Crises in some countries. Archives)	Descriptive analytical study	The natural crises national information systems of improve cooperation, information exchange and coordination in the management of natural crises through providing methods, terminology, information formats and standard operating procedures.
24	Ahmadi and Manoochehri (2020)	Assessing the status and analysis of factors affecting the desirability of crisis management of environmental hazards in Ghayenat city	2020 (Iran)	Descriptive analytical study (*n* = 100)	The crisis management situation in Ghaenat city was not satisfactory because of weakness in planning, implementation and supervision of city crisis management, social deficiencies, weakness in legislation and policy and weakness in education and practical skills.

### Disaster education

The results of training programmes by Najafi Ghezeljeh et al. (2019), Ahmadi and Manoochehri (2020), Tint et al. (2015), Ahadpourmarin and Shahrakipour (2013), Nakhaei, Tabiee and Saadatjou (2019) and Heidarnia et al. (2016) indicate that the pattern of education and building trust for community participation varies depending on the social, indigenous and cultural characteristics of each country.

According to these researches, training is one of the most successful ways to raise the production index in human societies and healthcare volunteers are useful for coordinating and helping those who are impacted.

### Disaster preparedness

Azmi, Masoum Poor and Shahmoradi (2017), Ostadtaghizadeh, Soleimani and Ardalan (2016), Kaveh-Firouz, Mahalati and Noorolahi (2009), Motieei Langroudi et al. (2015), and Patel, Kermanshachi and Namian (2020) all placed an emphasis on disaster preparedness. People who are impacted by natural disasters may suffer damages.

Therefore, catastrophe preparedness and public education are crucial. Planning and training are cited as being essential for disaster preparedness by Seyedin, Sohrabi Zadeh and Zaboli (2019), Porfande and Shoqi (2016) and Lee and Lee (2019).

### Disaster information

Through the evaluation of communication networks and how people perceive disasters, Jahangiri, Sadighi and Hedayati (2014), Asghari Zamani and Babaie Aqdam (2016) and Hayavi Haghighi, Moghaddasi and Rabiei (2019) investigated the appropriate methods and strategies for transferring information about defending against disaster to prepare people for natural disasters.

## Discussion

The aim of this systematic review was to learn more about the variables influencing Iranian healthcare volunteers’ preparation for natural disasters. Similar to Khalili et al. (2018), the education concept placed a strong emphasis on people’s involvement at the neighbourhood and local level in urban and rural areas. A community-based education strategy to disaster preparedness was used to implement this education and mobilising of people. According to Jahangiri et al. (2014), notification should not alter people’s views or behaviours; rather, it should motivate them to take preventive measures while raising their awareness of the risk of earthquakes. The community’s chosen strategies and each region’s indigenous culture should be taken into consideration while selecting notification and teaching materials. It is critical to consider how the public school system uses mass media, particularly radio and television. One of the key aspects of health management in catastrophes, according to Nakhaei et al. (2019), is strengthening healthcare workers’ knowledge, awareness and preparedness. In a study on disaster preparedness, Kolivand et al. (2021) showed that optimisation tools could enhance organisational performance. Training sessions ought to be offered to encourage optimisation in order to boost catastrophe management effectiveness. Another measure that has been suggested to improve disaster risk management is holding festivals and sharing successful experiences and accomplishments, as well as increasing the productivity of Iranian universities, recognising exceptional performance at the local, national and international levels, improving understanding, encouraging innovation, assessing people’s needs, promoting motivation, implementing rapid management and developing goals and executive plans.

One of the objectives of sustainable development is to increase resilience and prepare people for natural disasters. Two categories of factors were used to categorise those influencing disaster preparation training:

- The techniques for making people more prepared.
- The role of training and experience in fostering preparedness, as examined by Farajzadeh et al. (2017).

Their research revealed a connection between management experience, performance and gender as well as knowledge and age. The intermediate and unacceptable readiness score for nurses demonstrated the need to increase the conditions and motivation for disaster preparedness. Additionally, Patel et al. (2020) highlighted the need of educating vulnerable populations on how to deal with catastrophes and showed a substantial link between a person’s place of residence and ethnicity in terms of training for disaster risk reduction. The findings of Patel et al. (2020) are in accordance with this study. Tehran’s districts 4, 5, 15 and 20 are high-priority locations where vulnerable groups must be strengthened through capacity building. It was discovered that population groups’ vulnerability is affected by both their vulnerability and their gender. Disasters have varied effects on men and women, and they have a higher negative effect on women and children than on males. Natural disasters affect both men and women differently depending on their social and economic status. Gender consideration is crucial for lowering risks and responding to disasters. In addition to gender, marital status also has an impact on a person’s level of susceptibility. Despite the vulnerability of women, a different perspective was stated in this study that saw women as a key player in the community’s empowerment to control risk. Women are mentioned as a ‘factor of change’ in this study, which is a pro for macroplanning that empowers cities (Lee & Lee 2019).

One strategy to lessen the harm caused by natural catastrophes, according to the study by Kaveh-Firouz et al. (2009), was increased public preparedness. Planning in this regard necessitates evaluating the attitude and level of awareness people have towards disasters. In this study, just 2.9% of respondents claimed to have knowledge of disaster management and safety guidelines as well as ideas for catastrophe prevention. The findings highlight the importance of public engagement in minimising irreversible harm while dealing with disasters by demonstrating the belief in participation in both groups of villagers and officials. Staff training and strategies to enhance the disaster management system and preparedness efforts were mentioned by Seyedin et al. (2019). According to the study by Porfande and Shoqi (2016), nursing pioneers play a crucial role before, during and after disasters. It was also discovered that taking disaster management courses helped nurses change their knowledge, attitude and practice. We discovered after a thorough assessment of earlier studies that the implementation phase does not include the three components of education, preparedness and information prior to disasters. People lack the appropriate level of awareness, particularly when it comes to new and sophisticated concepts.

The general populace does not receive ongoing education. The purpose of this study was to look into methods for encouraging people to take preventative measures and engage in healthy activities and to set the right environment for such behaviour.

Unfortunately, this question has been left unanswered in earlier research, and a review of these studies has only partially addressed the researcher’s concerns. As a result, despite several studies, there is still much to be discovered about disaster education.

On the other side, there are a range of natural calamities, including quakes, floods, storms, pandemics like COVID-19, etc., that cause a variety of physical, social, cultural and biological losses. Just the natural disasters were studied in this study.

As we were only able to use four researchers to search the articles, there may be some bias in the findings. To develop training programs and gather instructional materials for disaster preparedness, more research is advised.

In organising training, it is advised that training objectives and vision statements be given top importance and that paying close regard to academic standards is essential.

Therefore, it is quite efficient to use professionals and specialists with experience in training requirements assessment to lessen the effects of disasters. Training initiatives created to lessen the effects of disasters must be actively carried out. In this regard, Ghanbari et al. (2011) investigated the effect of holding training courses and maneuvers on the preparation of nurses in coping with natural disasters in Iran. Their findings showed a considerable increase in the scores of knowledge, attitude and performance in the subjects of the study by Ghanbari et al. (2011). The studies by Heydari and Arabshahi (2005), Wang et al. (2008), Bartley, Fisher and Stella (2007), and Idrose et al. (2007) confirmed the effective role of holding maneuvers and training courses in the preparation of healthcare volunteers in coping with disasters. The preparation and ability to respond to the needs created during a disaster are directly under influence of people’s previous training and experiences. The findings by Fung, Loke and Lai (2008), Nasrabadi et al. (2007), Katz et al. (2006) and Duong (2009) showed that a large percentage of healthcare volunteers and nurses, who did not receive training, did not react well in coping with disasters. Also, Hussar (2006) states the considerable effect of educational programs on the reduction of death rate in injured people during the disaster. Therefore, there is no proper planning in place, and no proper planning for the education of people and healthcare volunteers in disasters in many countries, as revealed by Khankeh, Mohhamadi and Ahmadi (2006).

Repeating maneuvers helps people become more skilled and prepared, which in turn helps them react and make decisions faster in an emergency situation. As a result, it is advised that intervention training programs be continued.

## Conclusion

Preparation and raising public awareness are the most crucial aspects of disaster management. Most people should be able to ‘learn’ about disaster risks and disaster prevention measures because of the training. The most effective substitute for going through a disaster to improve public preparation is simulated learning.
