# Erratum: Determinants of health volunteer training in natural hazard management in Iran

**DOI:** 10.4102/jamba.v16i1.1562

**Published:** 2024-01-11

**Authors:** Fereshteh F. Amini, Alireza A. Hidarnia, Fazlollah F. Ghofranipour, Mohammad E. Motlagh, Abdul Majid RahPima, Navvab Shamspour

**Affiliations:** 1 Department of Medical Surgical, School of Nursing and Midwifery, Tehran University of Medical Sciences, Tehran, Islamic Republic of Iran; 2 Health Education and Health Promotion, Faculty of Medical Sciences, Tarbiat Modares University, Tehran, Islamic Republic of Iran; 3 Department of Health Education and Health Promotion, Faculty of Medical Sciences, Tarbiat Modares University, Tehran, Islamic Republic of Iran; 4 Department of Community Medicine, School of Medicine, Ahvaz Jundishapur University of Medical Sciences, Ahvaz, Islamic Republic of Iran; 5 Department of Public Policy, Islamic Azad University, Tehran Branch, Iran; 6 Research Center for Emergency and Disaster Resilience, Red Crescent Society of Islamic Republic of Iran, Tehran, Islamic Republic of Iran

In the published article, Amini, F.F., Hidarnia, A.A., Ghofranipour, F.F., Motlagh, M.E., RahPima, A.J. & Shamspour, N., 2023, ‘Determinants of health volunteer training in natural hazard management in Iran’, *Jàmbá: Journal of Disaster Risk Studies* 15(1), a1384. https://doi.org/10.4102/jamba.v15i1.1384, there was an error in the corresponding author’s full name and email address. The full name and email address was given incorrectly as Fereshteh Amini, fereshtehamini@modares.ac.ir. The correct full name and email address should be Alireza Hidarnia, hidarnia@modares.ac.ir.

The publisher apologises for this error. The correction does not change the study’s findings of significance or overall interpretation of the study’s results or the scientific conclusions of the article in any way.

